# StabFL: Stabilized Logit Adjustment with Queue-Augmented Representation Learning for Label-Skewed Federated Learning

**DOI:** 10.3390/s26196033

**Published:** 2026-09-23

**Authors:** Yujin Shin, Suhan Choi, JaeYeon Park

**Affiliations:** 1 School of Integrated Technology, College of Computing, Yonsei University, Seoul 03722, Republic of Korea; yujin_shin@yonsei.ac.kr; 2 Department of Mobile Systems Engineering, PRIMUS International College, Yongin 16890, Republic of Korea

**Keywords:** federated learning, label skew, logit adjustment, proximal regularization, supervised contrastive learning, feature queue

## Abstract

Federated learning under label skew aggregates models trained with different local class distributions. Local logit adjustment can reduce class-dependent bias, but the adjusted loss updates both the encoder and classifier in client-specific directions. ***StabFL*** optimizes a joint local objective that combines logit-adjusted classification loss, full-model proximal regularization, and supervised contrastive learning with a client-local queue. The queue retains recent labeled projected representations and extends the comparison set available to small mini-batches. Across CIFAR-10 and PAMAP2, ***StabFL*** reaches 85.57±1.36% and 75.76±3.21% accuracy on CIFAR-10 under moderate and severe label skew, and 83.21±0.46% and 74.68±4.10% on PAMAP2 under the same skew levels. Compared with ***StabFL*** w/o Queue, the client-local queue raises mean accuracy by 0.39 and 1.66 percentage points on CIFAR-10 and by 1.76 and 4.44 percentage points on PAMAP2 while transmitting no queued projected representations, labels, or local class priors.

## 1. Introduction

Federated learning trains a shared model while data remain on distributed clients [1,2,3,4]. In mobile and wearable sensing, each client often records sensor data from a user whose routines, activities, and recording schedules determine which classes are observed locally [5,6,7,8]. So, the resulting class distributions can differ substantially across clients. Partial participation changes the set of local distributions represented in each communication round while limited client resources often require small local mini-batches. These conditions affect both classification and representation learning. Label skew biases local optimization toward locally frequent classes while small mini-batches provide only a limited set of comparisons for representation learning. Addressing these limitations also requires attention to green computing in sensor-enabled systems, where battery-powered mobile and wearable clients have limited memory and computational resources, and both local training and wireless model exchange consume energy. Therefore, improvements to local classification and representation learning should be evaluated alongside the additional computation, storage, and communication they require.

For client-specific classification bias, local logit adjustment incorporates the local class prior (i.e., each client’s class distribution) into the classification loss [9,10,11]. The adjustment changes class-dependent gradients and can reduce the bias toward locally frequent classes. Those gradients update both the feature encoder and classifier, and the adjustment differs across clients because their class priors differ. Therefore, the locally updated models may move in different directions from one another before server aggregation. Federated optimization methods such as FedProx [12], SCAFFOLD [13], FedDyn [14], and FedDFPA [15] control local updates under heterogeneous data, but they do not determine which trainable components should be constrained when the classification loss uses a client-specific logit adjustment. A classifier-only constraint leaves the feature basis produced by the feature encoder less constrained. This motivates proximal regularization over the complete trainable model.

Full-model proximal regularization addresses the stability of the adjusted local update, but local logit adjustment remains a classification objective. Supervised contrastive learning (SupCon) [16] can provide an additional training signal for the feature encoder by attracting samples from the same class. Its usefulness depends on the comparison set available to each anchor. With batch-only SupCon, an anchor receives no positive comparison when the current mini-batch contains no other sample from its class. This limitation is common for minority classes in small, label-skewed mini-batches.

We propose ***StabFL***, which combines these mechanisms in a joint local objective. Each selected client first computes its local class prior and applies logit adjustment to the classification loss. It then regularizes the entire trainable model, including the encoder, projection head, and classifier, relative to the received global model. In addition, the client evaluates supervised contrastive regularization using both the current mini-batch and a persistent client-local FIFO queue. This queue stores labeled projected representations generated from preceding local mini-batches and makes them available as additional contrastive comparisons in subsequent updates while keeping the queued representations and their labels on each client. On both CIFAR-10 and PAMAP2, it raises the fraction of anchors with at least one same-class comparison, which we call valid anchors, to nearly 100% and increases the mean number of positives per valid anchor by more than an order of magnitude, for example from 2.54 to 77.85 and from 4.00 to 138.01 under moderate and severe skew on CIFAR-10.

The design principle of ***StabFL*** is to jointly stabilize client-specific learning updates and enrich the local contrastive comparison set. Although logit adjustment, proximal regularization, and cross-batch representation reuse are established mechanisms, the novel features of ***StabFL*** concern their coordinated use under label skew, limited mini-batch comparisons, and intermittent client participation. The classification and contrastive losses share the encoder but involve different heads, motivating full-model proximal regularization across the trainable components of the joint objective. The persistent client-local queue complements this parameter-level constraint by supplying historical same-class comparisons without transmitting its contents. We evaluate this design through proximal-scope, queue, and component-interaction analyses, examining when the components provide complementary benefits rather than assuming that their effects are independent.

We evaluate ***StabFL*** on CIFAR-10 image classification and PAMAP2 wearable activity recognition under moderate and severe label skew, partial and full client participation, and two local batch sizes. The main comparison includes FedAvg, FedProx, MOON, FedLC, and FedRCL. ***StabFL*** reaches the highest mean accuracy among these methods in all four conditions, with 85.57±1.36% and 75.76±3.21% on CIFAR-10 under moderate and severe skew, and 83.21±0.46% and 74.68±4.10% on PAMAP2 under the same skew levels. The variant comparisons show that the benefit of local logit adjustment depends strongly on proximal scope under severe skew. They also show that the client-local queue raises the mean accuracy over ***StabFL*** without Queue by 0.39 and 1.66 percentage points on CIFAR-10 and by 1.76 and 4.44 percentage points on PAMAP2 under moderate and severe skew, respectively.

Our contributions are summarized as follows.

- We show that coupling local logit adjustment with full-model proximal regularization stabilizes local updates and improves accuracy especially under severe label skew.
- We introduce a persistent client-local FIFO queue of labeled projected representations, integrated into the stabilized local objective to extend supervised contrastive comparisons beyond the current mini-batch without transmitting the queue contents.
- We evaluate ***StabFL*** across image classification and wearable activity recognition while varying label skew, client participation, and local batch size. The comparisons examine component effects and interactions, proximal scope, and the contribution of the client-local queue.

## 2. Related Work

### 2.1. Federated Optimization Under Heterogeneous Data

FedAvg [1] trains selected clients on their local objectives for multiple steps and aggregates the returned parameters. When client distributions differ, local gradients can point in different directions causing the aggregated update to deviate from the descent direction of the global objective. FedProx [12] adds a proximal penalty between each local model and the received global model. SCAFFOLD [13] uses client and server control variates to correct gradient bias during local training. FedDyn [14] adds a dynamic linear term to reduce inconsistency between local and global optimization across rounds. These methods control the magnitude or direction of local updates before aggregation.

The same mechanisms do not directly remove class-dependent bias from a client-specific label distribution. A proximal regularization can keep local parameters near the global model while the classification loss remains dominated by locally frequent classes. Therefore, optimization stability and local class-prior correction address different parts of the local update.

### 2.2. Local Logit Adjustment Under Label Skew

Class-imbalance methods use class frequencies to modify the classification loss or decision scores. Balanced Softmax inserts class counts into softmax normalization [17]. Logit adjustment adds a prior-dependent term to each class logit during training or inference [9]. In federated learning, FedRS [10] restricts logits for classes that are absent or rarely observed on a client. FedLC [11] uses client-level class occurrence probabilities to calibrate local logits before computing the classification loss. These methods alter the class-specific gradients produced by a skewed client dataset.

A client-specific adjustment also changes the model update that is later aggregated by the server. Different local class priors produce different adjusted gradients, and those gradients update both the encoder and classifier. Existing logit-correction methods do not examine how the proximal scope affects this coupled update. A classifier-only constraint can keep the final classifier layer near its global reference while the encoder continues to change under the adjusted loss. The appropriate proximal scope for local logit adjustment therefore remains an empirical design question.

### 2.3. Federated Contrastive Learning with Limited Local Comparisons

Federated representation methods reduce the effects of client heterogeneity through feature constraints or class-level information. FedBABU [18] trains a shared feature extractor while reserving the classifier for local adaptation. MOON [19] uses model-level contrast to keep local representations close to those of the current global model and separate them from previous local states. FedProto [20] communicates class prototypes and aligns local features with aggregated prototypes, while FedPCL [21] performs prototype-wise contrastive learning using representations from pretrained models. FedRCL [22] uses relaxed sample-level contrastive learning that penalizes excessively similar within-class pairs to reduce representation collapse under heterogeneous data. DCFL [23] separates alignment and uniformity terms to account for the finite samples available on each client.

These methods guide representation learning through model-level contrast, prototypes, or modified contrastive objectives. The comparison set available to each anchor is another important consideration. Batch-only SupCon requires another same-class sample in the current mini-batch for an anchor to contribute [16]. Memory-based learning extends comparisons across mini-batches. For example, Cross-Batch Memory [24] reuses previously computed embeddings for supervised metric learning. Therefore, cross-batch representation reuse is an established mechanism. ***StabFL*** incorporates this mechanism through a client-local FIFO queue of labeled projected representations. Each queue persists across communication rounds, remains unchanged during non-participation, and is conditionally cleared according to encoder-parameter similarity when the client returns. Its contents provide additional positive and negative comparisons without transmitting the stored representations or labels.

The design principle of ***StabFL*** is to combine this expanded comparison set with stabilized, client-specific classification updates. Unlike MOON’s model-level contrast, ***StabFL*** uses label-defined sample-level comparisons. While FedRCL modifies within-class interactions to preserve representation diversity, ***StabFL*** extends comparisons beyond the current mini-batch and integrates them with local logit adjustment and full-model proximal regularization. The classification loss updates the encoder and classifier, while the contrastive loss updates the encoder and projection head. We apply proximal regularization to all three components to constrain their deviation from the received global model during joint local training.

Table 1 compares the main objectives, treatment of label skew, communicated information, client-local memory, and computational requirements of the related methods. Table 2 then summarizes four key design elements, including prior correction, proximal constraints, contrastive learning, and cross-batch memory. ***StabFL*** uses a log-prior adjustment distinct from FedLC’s calibration and adopts the full-model proximal regularization principle of FedProx. Its methodological distinction lies in coordinating these mechanisms with supervised sample-level contrast and a persistent client-local queue within a single local objective. Complete-method comparisons with FedLC and FedRCL evaluate overall performance, while the component ablations examine proximal scope, component interactions, and the contribution of the client-local queue. The computational overhead of ***StabFL*** is quantified in Section 6.5.

## 3. Problem Formulation

We consider local training under client-specific label distributions and small mini-batches. Local logit adjustment changes the gradients of both the encoder and classifier, so the resulting update must remain suitable for parameter averaging. Small mini-batches can also leave anchors without same-class comparisons for supervised contrastive learning.

### 3.1. Label-Skewed Federated Learning

Consider a federated learning system with *K* clients and *C* classes. Client *k* holds

(1) $$D_{k}={\left\{\left(x_{i},y_{i}\right)\right\}}_{i=1}^{n_{k}},$$

where $n_{k}$ is the number of local samples held by client (*k*).

At communication round *t*, the server selects a participating client set $S_{t}$ and broadcasts the current global model $w^{t}$. Each selected client initializes its local model with $w^{t}$, performs local training, and returns $w_{k}^{t+1}$. The server computes

(2) $$w^{t+1}=\sum_{k\in S_{t}}\frac{n_{k}}{\sum_{j\in S_{t}}n_{j}}w_{k}^{t+1}$$

Let $n_{k,c}$ denote the number of samples from class *c* on client *k*. The local class prior is

(3) $$\pi_{k,c}=\frac{n_{k,c}}{n_{k}}$$

Under label skew, $\pi_{k,c}$ varies across clients. Partial participation introduces additional round-to-round variation because changes in $S_{t}$ alter the mixture of local class priors represented in the aggregated update.

The predictive model contains a feature encoder $f_{\theta }$ and a classifier *W*. The encoder maps an input $x_{i}$ to a feature used by the classifier to compute the raw class logits

(4) $$s\left(x_{i}\right)=Wf_{\theta }\left(x_{i}\right)$$

For contrastive regularization, the model additionally contains a projection head $g_{\phi }$. It maps the same encoder feature to a normalized projected representation

(5) $$z_{i}=\frac{g_{\phi }\left(f_{\theta }\left(x_{i}\right)\right)}{{∥g_{\phi }\left(f_{\theta }\left(x_{i}\right)\right)∥}_{2}}$$

The classifier operates on the encoder feature, whereas the supervised contrastive loss compares the projected representations $z_{i}$. Both objectives update the shared encoder, and the client-local queue stores detached tuples of projected representation $z_{i}$, label $y_{i}$, and a client-local source identifier $r_{i}$.

### 3.2. Local Training Requirements

The first requirement concerns the compatibility of locally adjusted updates with global aggregation. Local logit adjustment changes the gradients of both the encoder and the classifier. A constraint applied only to the classifier can keep *W* close to its global reference while leaving $f_{\theta }$ less constrained under the adjusted classification objective. The features produced by the encoder may then become inconsistent with the constrained classifier. Stabilization should account for the encoder and classifier as coupled components of the local model.

The second requirement concerns positive comparisons for supervised contrastive learning. For anchor *i* in local mini-batch $B_{k}$, define

(6) $$P_{k}\left(i\right)=\left\{j\in B_{k}\setminus \left\{i\right\}|y_{j}=y_{i}\right\}$$

The anchor contributes to the supervised contrastive loss only when

(7) $$\left|P_{k}\left(i\right)\right|\;>\;0$$

We refer to an anchor that satisfies this condition as a valid anchor. Under label skew and small local mini-batches, infrequent classes can instead satisfy

(8) $$\left|P_{k}\left(i\right)\right|\;=\;0$$

Such samples provide no supervised contrastive signal during the corresponding local update. Changing the contrastive objective cannot recover same-class samples that are absent from the current mini-batch. The comparison set must extend beyond the current batch while keeping sample-derived representations and labels within the client.

These conditions define two coupled requirements for local training. The parameter updates produced by client-specific classification and contrastive learning should be stabilized across their shared encoder and respective heads. The supervised comparison set should also include additional same-class samples without cross-client exchange of representations or labels. Parameter regularization and comparison-set expansion address different limitations, and neither alone satisfies both requirements.

## 4. ***StabFL***

### 4.1. Overview

Figure 1 shows one communication round. The server sends $w^{t}$ to each selected client. A client estimates its class prior and evaluates a joint local objective containing logit-adjusted classification, full-model proximal regularization, and queue-augmented supervised contrastive regularization. After local training, the client uploads its model state, and the server applies Equation (2).

The three terms address complementary aspects of the same local update. Logit adjustment changes the class-dependent pressure of the classification loss. The proximal term controls the complete trainable model relative to the received state. The client-local queue supplies recent labeled projected representations that are unavailable in the current mini-batch.

### 4.2. Stabilizing Local Logit Adjustment

#### 4.2.1. Local Logit Adjustment

For client *k*, we compute the local class prior as

(9) $${\tilde{\pi }}_{k,c}=\frac{n_{k,c}+\epsilon }{n_{k}+C\epsilon }$$

where *C* is the number of classes and ϵ is a numerical-stability term.

Given raw logits $s\left(x\right)\in R^{C}$, the adjusted logits are

(10) $${\tilde{s}}_{k,c}\left(x\right)=s_{c}\left(x\right)+\sigma \log{\tilde{\pi }}_{k,c}$$

where σ≥0 controls the strength of logit adjustment.

For local mini-batch B, the logit-adjusted (LA) classification loss is

(11) $$L_{\mathrm{LA}}=-\frac{1}{\left|B\right|}\sum_{i\in B}\log\frac{\exp\left({\tilde{s}}_{k,y_{i}}\left(x_{i}\right)\right)}{\sum_{c=1}^{C}\exp\left({\tilde{s}}_{k,c}\left(x_{i}\right)\right)}.$$

The adjustment changes the training loss without adding an inference module. The local class counts are neither included in the model state nor sent to the server.

#### 4.2.2. Full-Model Proximal Regularization

The client constrains its movement from the received global model with

(12) $$L_{\mathrm{prox}}=\frac{\mu }{2}{∥w_{k}-w^{t}∥}_{2}^{2}$$

The full-model scope contains the trainable encoder, projection head, and classifier parameters. The same coefficient is applied to all three components in ***StabFL***.

The scope follows the shared gradient paths of the local objective. The encoder changes the feature basis, the classifier adapts its boundaries within that basis, and the projection head maps the same features to the contrastive space. A classifier-only constraint can limit final-layer movement while leaving a larger encoder update. Constraining (θ,ϕ,W) together controls the complete trainable model during local optimization.

### 4.3. Improving Representation Learning with a Client-Local Queue

#### 4.3.1. Limits of Batch-Only Supervised Contrastive Learning

Let $z_{i}=g_{\phi }\left(f_{\theta }\left(x_{i}\right)\right)$ denote a normalized projected representation. In a mini-batch B, anchor *i* contributes to a supervised contrastive loss only when another sample has label $y_{i}$. A small, label-skewed mini-batch can therefore provide few valid anchors and few same-class comparisons.

#### 4.3.2. Supervised Contrastive Learning with the Queue

Each client maintains a FIFO queue $Q_{k}$ of tuples $\left({\bar{z}}_{j},y_{j},r_{j}\right)$ from recent mini-batches, where ${\bar{z}}_{j}$ is a detached projected representation, $y_{j}$ is its class label, and $r_{j}$ is a stable source identifier. The source identifier is assigned before data shuffling and remains unchanged when the same sample is encountered across local epochs or communication rounds. Current mini-batch samples act as anchors. Although representations from repeated encounters with the same source may remain in the queue, they are excluded whenever that source becomes the current anchor.

For anchor *i*, the comparison and positive sets are defined as

(13) $$\begin{array}{cc} A(i) & =\left\{a\in \left(\left(B\setminus \left\{i\right\}\right)\cup Q_{k}\right):r_{a}\neq r_{i}\right\}, \end{array}$$

(14) $$\begin{array}{cc} P(i) & =\left\{a\in A\left(i\right):y_{a}=y_{i}\right\}. \end{array}$$

Thus, an earlier representation of the current source sample is excluded from both the positive set and the contrastive denominator. For notational simplicity, $z_{a}$ below denotes the current projected representation when a∈B and the stored representation ${\bar{z}}_{a}$ when $a\in Q_{k}$.

The normalized comparison probability and queue-augmented loss are

(15) $$\begin{array}{cc} q_{i,a}^{Q} & =\frac{\exp(z_{i}^{⊤}z_{a}/\tau )}{\sum_{u\in A(i)}\exp\left(z_{i}^{⊤}z_{u}/\tau \right)} \end{array}$$

(16) $$\begin{array}{cc} L_{\mathrm{SupCon}}^{Q} & =-\frac{1}{\left|V\right|}\sum_{i\in V}\frac{1}{\left|P(i)\right|}\sum_{p\in P(i)}\log q_{i,p}^{Q} \end{array}$$

where V={i∈B:|P(i)|>0}.

Each client owns a separate queue. The queue is initialized empty at the beginning of a training run, persists across communication rounds, and remains unchanged while the client is not selected. When the client participates again, it reuses its existing queue unless the queue is reset. Queues are never shared or aggregated across clients. The source identifiers are used only for local comparison filtering and are never transmitted.

Let $\theta_{k}^{\mathrm{last}}$ denote the encoder parameter vector retained after client *k* completed its previous local training, and let $\theta^{t}$ denote the encoder parameter vector broadcast by the server in round *t*. When client *k* participates again, it computes

(17) $$\rho_{k}^{t}=\frac{{\left(\theta_{k}^{\mathrm{last}}\right)}^{⊤}\theta^{t}}{{∥\theta_{k}^{\mathrm{last}}∥}_{2}{∥\theta^{t}∥}_{2}}$$

The client clears $Q_{k}$ when $\rho_{k}^{t}<\delta$. No reset is performed on its first participation because no previous local encoder is available. After local training, the client retains its updated encoder parameter vector for the next comparison.

The encoder-parameter cosine rule is intended as a low-cost, data-free heuristic for detecting substantial changes between the encoder retained from the client’s previous participation and the encoder received in the current round. It requires neither an additional forward pass nor a separate reference batch. However, a high parameter cosine similarity does not guarantee functional equivalence or compatibility between current and queued representations. Equation (17) should therefore be interpreted as a practical safeguard rather than a formal measure of representation freshness. Section 6.4 compares alternative reset strategies, parameter scopes, and threshold values.

Once the reset decision has been made, the client proceeds with local training. For each mini-batch, it computes the current projections, forms the source-filtered comparison and positive sets, and evaluates the complete local objective before updating the model parameters. After the optimizer step, each current projection $z_{i}$ is detached and stored in the queue together with its label $y_{i}$ and source identifier $r_{i}$. The oldest entries are removed when the capacity exceeds *M*. Queue entries, labels, and source identifiers remain local and are not aggregated or transmitted. After completing local training, the client retains its updated encoder parameter vector as $\theta_{k}^{\mathrm{last}}$ for the next comparison.

Note that the projection head is used only for the supervised contrastive objective during training. It is included in the federated model state and aggregation during training, but is discarded at inference, where predictions are produced using only the encoder and classifier.

### 4.4. Joint Local Training and Aggregation

The final client objective is

(18) $$L_{k}=L_{\mathrm{LA}}+L_{\mathrm{prox}}+\lambda L_{\mathrm{SupCon}}^{Q}$$

where λ balances the queue-augmented supervised contrastive loss against the classification and proximal terms.

The interaction among the terms can be made explicit through their parameter-wise gradients. Let (θ,ϕ,W) denote the current client parameters and $\left(\theta^{t},\phi^{t},W^{t}\right)$ the corresponding parameters of the received global model. With queued representations treated as detached local state, the gradients are

(19) $$\begin{array}{cc} \nabla_{\theta }L_{k} & =\nabla_{\theta }L_{\mathrm{LA}}+\lambda \nabla_{\theta }L_{\mathrm{SupCon}}^{Q}+\mu \left(\theta -\theta^{t}\right), \end{array}$$

(20) $$\begin{array}{cc} \nabla_{W}L_{k} & =\nabla_{W}L_{\mathrm{LA}}+\mu \left(W-W^{t}\right), \end{array}$$

(21) $$\begin{array}{cc} \nabla_{\phi }L_{k} & =\lambda \nabla_{\phi }L_{\mathrm{SupCon}}^{Q}+\mu \left(\phi -\phi^{t}\right). \end{array}$$

These expressions describe the gradients of the joint objective, rather than an optimizer-specific update rule.

The classification and contrastive terms both affect the shared encoder, while the classifier and projection head participate in their respective objectives. A classifier-only proximal constraint therefore leaves the encoder and projection head without a direct proximal penalty. Full-model regularization extends this constraint to all trainable components, but it does not change the samples available for contrastive learning. Conversely, the queue changes the comparison set and thus the contrastive gradient, but does not itself constrain parameter deviation from the received global model.

The resulting design coordinates class-prior correction, parameter-level stabilization, and comparison-set expansion within the same local update. Local logit adjustment does not supply missing positive comparisons, and queue reuse does not replace class-prior correction or proximal regularization. These distinct roles motivate the joint design without implying that the combination must improve every setting. Section 6.3.2 examines the configuration-dependent effects of the components, while Section 6.3.1 isolates the queue contribution through the matched batch-only comparison.

Persistent memory also introduces a limitation that parameter regularization alone does not resolve. Stored projections were generated by earlier encoder and projection-head states, whereas the proximal reference is the current global model. Proximity to that reference does not guarantee compatibility with all queued representations. The encoder-similarity-based reset rule in Equation (17) therefore provides a heuristic safeguard against model mismatch rather than a formal guarantee of representation freshness.

After local optimization, each participating client returns its updated model state, and the server aggregates the returned parameters using Equation (2). The queue and local class priors remain on the client. Algorithm 1 summarizes the client and server operations.
**Algorithm 1** Federated training with stabilized logit adjustment and a client-local queue**Require:** Initial model $w^{0}$, rounds *T*, local epochs *E*, queue capacity *M*, reset threshold δ  1:**for** t=0,…,T−1 **do**  2:       Server samples $S_{t}$ and broadcasts $w^{t}$  3:       **for all** $k\in S_{t}$ **in parallel do**  4:             Set $w_{k}\leftarrow w^{t}$ and compute ${\tilde{\pi }}_{k}$  5:             **if** client *k* has not participated before **then**  6:                   Initialize $Q_{k}\leftarrow \emptyset$  7:             **else**  8:                   Compute $\rho_{k}^{t}$ using Equation (17)  9:                   **if** $\rho_{k}^{t}<\delta$ **then**10:                         Clear $Q_{k}$11:                   **end if**12:             **end if**13:             **for** e=1,…,E **do**14:                   **for all** mini-batches $B=\left\{\left(x_{i},y_{i},r_{i}\right)\right\}\subset D_{k}$ **do**15:                         Compute current projections16:                         Form A(i) and P(i) by excluding entries with $r_{a}=r_{i}$17:                         Compute $L_{\mathrm{LA}}$, $L_{\mathrm{prox}}$, and $L_{\mathrm{SupCon}}^{Q}$18:                         Take one optimizer step on Equation (18)19:                         Enqueue $\left(z_{i},y_{i},r_{i}\right)$ after detaching $z_{i}$20:                         Remove the oldest entries if $|Q_{k}|\;>\;M$21:                   **end for**22:             **end for**23:             Retain the updated encoder as $\theta_{k}^{\mathrm{last}}$24:             Upload $w_{k}$ to the server25:       **end for**26:       Aggregate $w^{t+1}$ using Equation (2)27:**end for**

### 4.5. Communication and Local Storage

For the model architecture used in our experiments, the queue, local class prior, and sample-derived projected representations are excluded from the transmitted state. A queue with capacity *M*, projected representation dimension *d*, and scalar width *b* uses approximately Mdb bytes for representations, with a small additional label array. Under the settings in Section 5, the queue tensors use 132 KiB per client.

## 5. Experimental Setup

The experiments examine whether full-model proximal regularization supports local logit adjustment and whether a client-local queue supplies same-class comparisons missing from small mini-batches. We study image classification and wearable activity recognition under different levels of label skew, client participation, and local batch size. All results are reported as mean ± standard deviation over five independent seeds. Within each seed, compared methods use the same client partition, participation sequence, and model initialization.

### 5.1. Datasets and Client Construction

CIFAR-10 contains 50,000 training images and 10,000 test images from ten classes [25]. We use 45,000 samples for client training and 5000 for validation, and construct 20 clients with a Dirichlet partition. The moderate and severe settings use α=0.3 and α=0.1, respectively.

PAMAP2 contains wearable sensor recordings of physical activities [26]. We retain 12 activities and use 18 channels sampled at 100 Hz. Each input is a 128-sample window with stride 64. We use a subject-independent split, holding out one subject for evaluation and using the remaining eight subjects for training and validation. The training windows are partitioned across 20 clients with α=0.3 for the moderate setting and α=0.1 for the severe setting.

Unless stated otherwise, five of the 20 clients participate in each communication round. The participation analysis additionally evaluates all 20 clients. Table 3 summarizes the remaining dataset and training settings.

Under severe label skew (α=0.1), clients contain 3.27±1.54 of 10 classes on CIFAR-10 and 3.00±1.52 of 12 activities on PAMAP2 on average. Across the evaluated partitions, an average of 134.6/200 and 180.0/240 client–class pairs, respectively, are empty, while no client is completely empty. All clients contain sufficient local data for the stated training configuration: the minimum training-set sizes are 102 samples for CIFAR-10, 70 windows for synthetic PAMAP2, and 78 windows for natural-client PAMAP2, all exceeding the batch size of 8.

For CIFAR-10, we use a compact ResNet8-type encoder with a 32-channel stem and three residual stages producing 64, 128, and 128 channels, followed by global average pooling to a 128-dimensional feature. For PAMAP2, we use a three-layer 1D CNN with 64, 128, and 128 output channels, batch normalization, ReLU activations, and temporal average pooling. The classifier is a single linear layer. For contrastive learning, both datasets use a two-layer 128→128→128 projection head with a ReLU activation and L2-normalized output.

For PAMAP2, we use the accelerometer and gyroscope channels from the hand, chest, and ankle, giving 18 input channels. Missing values are interpolated along the temporal dimension, and each channel is standardized using statistics computed from the non-test training subjects. Windows are constructed within homogeneous activity segments so that they do not cross activity boundaries, and each window inherits the activity label of its segment.

### 5.2. Baselines and Method Variants

The main baseline comparison includes FedAvg, FedProx, MOON [19], FedLC [11], FedRCL [22], and ***StabFL***. FedProx uses a coefficient of 0.003 on CIFAR-10 and 0.01 on PAMAP2. MOON uses standard cross-entropy with its model-contrastive loss. Its contrastive coefficient is 1.0 and its temperature is 0.5. Each client uses its previous local model as the negative reference, with the round-start global model used on the client’s first participation.

FedLC provides a direct comparison with class-frequency-dependent logit calibration under label skew. FedRCL provides a recent sample-level contrastive baseline that penalizes excessively similar within-class pairs to reduce representation collapse under heterogeneous data. These baselines broaden the comparison beyond proximal stabilization and model-level contrast. Standalone FedLC is evaluated without the additional proximal term used in the FedLC + Prox variant.

A separate comparison examines FedLC + Prox, logit adjustment (LA) + Prox, ***StabFL*** w/o Queue, and ***StabFL***. The Prox suffix denotes the full-model proximal term with μ=0.001 used by ***StabFL***, while the standalone FedProx baseline uses the dataset-specific coefficients above. FedLC + Prox combines FedLC with this matched proximal term. LA + Prox uses the logit-adjusted classification loss and the same proximal term. ***StabFL*** w/o Queue keeps the logit adjustment, projection head, and supervised contrastive weight of ***StabFL***, but its contrastive comparison set contains only the current mini-batch.

The first analysis compares cross-entropy and LA with and without full-model proximal regularization. The proximal-scope analysis compares classifier-only, split, and full-model constraints. The queue analysis holds LA + Prox fixed and compares no contrastive term, batch-only SupCon, and supervised contrastive learning with the client-local queue. The classifier-only scope penalizes only the classifier weights and bias with μ=0.003. The split scope applies μ=0.001 to the classifier and μ=0.0003 to all remaining trainable parameters, while the full-model scope applies a single coefficient of μ=0.001 to the classifier, encoder, and projection head. Each scope uses its best-performing coefficient configuration from a preliminary screening.

### 5.3. Training Details and Metrics

Hyperparameter and checkpoint selection use validation data only. For CIFAR-10, approximately 10% of the official training pool is reserved for client validation; PAMAP2 uses a 20% client-local validation split. We pool the client validation predictions and use macro-F1 as the selection metric. Hyperparameter screening uses a single seed, whereas the headline evaluation uses five seeds. The reported checkpoint is the communication round with the highest pooled-validation macro-F1, and the test sets are not used for hyperparameter or checkpoint selection. A single ***StabFL*** configuration was selected through this validation-based screening and fixed across CIFAR-10 and PAMAP2, whereas dataset-specific baseline coefficients were selected using the same validation-only procedure.

All methods are trained with Adam using a learning rate of $10^{-3}$, weight decay of $10^{-4}$, and five local epochs per communication round. Batch-normalization affine parameters and running statistics are included in the global model state and aggregated across participating clients. We do not maintain client-specific batch-normalization statistics as in FedBN. ***StabFL*** uses σ=1, μ=0.001, λ=1, τ=0.1, queue capacity M=256, and reset threshold δ=0.95. The class-prior smoothing constant is $\epsilon =10^{-8}$. CIFAR-10 and PAMAP2 run for 100 rounds under moderate skew and 200 rounds under severe skew. We report global test accuracy and macro-F1 as mean ± standard deviation over the five independent seeds. Macro-F1 assigns equal weight to the class-wise F1 scores and therefore provides an imbalance-aware complement to overall accuracy. For the proximal-scope analysis, we additionally record how far each participating client moves from the received global model. After local training in each round, we measure the L2 distance between the local parameters and the round-start global parameters, separately for the classifier weights and bias (classifier drift) and for the encoder parameters (encoder update norm). Both are dimensionless parameter-space norms averaged over participating clients, communication rounds. Their absolute magnitudes are not directly comparable because the encoder contains far more parameters. All methods were implemented in PyTorch 2.5.1 and executed on a server with an Intel Core i9-9900K CPU, 64 GB RAM, and two NVIDIA GeForce RTX 3090 GPUs.

## 6. Evaluation

### 6.1. Overall Performance

We first compare the global performance of the evaluated methods in the default partial-participation setting, where five of the 20 clients are selected in each communication round. Figure 2 presents the macro-F1 scores of FedAvg, FedProx, MOON, FedLC, FedRCL, ***StabFL*** w/o Queue, and ***StabFL*** on both datasets under moderate and severe label skew. Table 4 additionally reports the exact accuracy and macro-F1 values under both moderate and severe label skew.

Specifically, Figure 2 shows that ***StabFL*** and its queue-free variant achieve the two highest mean macro-F1 scores in the moderate-skew settings. Under severe skew, ***StabFL*** achieves the highest mean macro-F1 on both datasets. The differences between the methods also become more pronounced under severe skew especially on CIFAR-10.

Table 4 provides the numerical results under severe skew. On CIFAR-10, ***StabFL*** achieves a mean macro-F1 of 75.84%, compared with 69.70% for FedProx and 71.31% for ***StabFL*** w/o Queue. The corresponding differences are 6.14 and 4.53 percentage points. On PAMAP2, ***StabFL*** reaches a mean macro-F1 of 72.82%, which is 0.45 percentage points higher than FedProx and 5.88 percentage points higher than ***StabFL*** w/o Queue.

The accuracy results show a similar pattern. ***StabFL*** achieves the highest mean accuracy on both datasets under severe skew, reaching 75.76% on CIFAR-10 and 74.68% on PAMAP2. Although these results compare the complete methods under the same experimental settings, they do not isolate the contribution of each component. Section 6.2 and Section 6.3 therefore examine the effects of proximal regularization, proximal scope, and the client-local queue in greater detail.

Natural subject-boundary evaluation. The Dirichlet construction provides controlled label-skew conditions and a consistent 20-client setting across CIFAR-10 and PAMAP2, but it does not preserve the natural subject boundaries of the wearable dataset. To complement this controlled setting, we conduct a subject-as-client evaluation using Leave-One-Subject-Out-Cross-Validation. The nine PAMAP2 subjects, 101 through 109, define the natural subject boundaries. In each fold, one subject is held out for testing, while each of the remaining eight subjects forms one federated client containing only that subject’s sensor windows. We report performance on the held-out subject and summarize the results across the nine folds.

As shown in Table 5, ***StabFL*** achieves the highest mean macro-F1 of 85.33%. It exceeds FedAvg, which records the highest competing mean, by 2.74 percentage points and FedProx by 2.88 percentage points. It also exceeds the ***StabFL*** w/o Queue variant by 3.40 percentage points. These results show that the highest mean performance of ***StabFL*** is also observed when natural subject boundaries are preserved and the test subject is excluded from all federated training clients.

### 6.2. Proximal Regularization and Scope

We first test whether proximal regularization simply improves any local objective or is especially useful when local logit adjustment is applied. Figure 3a compares cross-entropy and logit adjustment (LA) with and without the same full-model proximal term on CIFAR-10. Under moderate skew, proximal regularization improves cross-entropy by 3.61 percentage points and LA by 4.77 percentage points. Under severe skew, the gains increase to 10.30 and 19.02 percentage points. The severe-skew gain is therefore 8.72 percentage points larger with LA, showing that the adjusted objective depends more strongly on controlling the local update.

Having established this interaction, we next ask which part of the local model should be constrained. Figure 3b shows little separation among the three scopes under moderate skew. Full-model regularization reaches 85.26%, while the classifier-only and split scopes reach 84.93% and 84.67%. The difference becomes clearer under severe skew. Full-model regularization reaches 74.21% and exceeds the split and classifier-only scopes by 2.31 and 2.72 percentage points.

Figure 3c helps explain this result. The classifier-only scope produces the smallest classifier drift, but its severe-skew encoder update norm is 13.92. Full-model regularization allows more classifier movement while reducing the encoder update norm to 9.01, and it gives the highest accuracy in Figure 3b. Limiting the classifier alone therefore does not sufficiently control the encoder update produced by the adjusted local loss.

To separate the effect of the regularized parameter set from that of the proximal coefficient, Table 6 compares classifier-only, encoder-and-projector, and full-model regularization using the same μ values. Full-model regularization achieves the highest mean macro-F1 at all three shared coefficients. This result suggests that the stronger performance of full-model regularization is not solely due to the choice of μ.

### 6.3. Ablation Study

#### 6.3.1. Queue Contribution

We next isolate the contribution of the client-local queue. Figure 4a compares LA + Prox, ***StabFL*** w/o Queue, and ***StabFL*** under partial participation. LA + Prox has no contrastive term. ***StabFL*** w/o Queue adds supervised contrastive learning using only the current mini-batch, while ***StabFL*** extends the same objective with the client-local queue.

The batch-only contrastive term does not improve LA + Prox consistently. It changes CIFAR-10 accuracy by less than 0.12 percentage points at either skew level, improves moderate-skew PAMAP2 by 1.42 percentage points, and reduces severe-skew PAMAP2 by 1.31 percentage points. Adding the queue raises the mean over ***StabFL*** w/o Queue in every condition. The gains are 0.39 and 1.66 percentage points on CIFAR-10 and 1.76 and 4.44 points on PAMAP2 under moderate and severe skew, respectively.

Figure 4b,c show what changes in the contrastive comparison set. Panel (b) reports the fraction of anchors with at least one same-class comparison, that is, the fraction of valid anchors. On CIFAR-10, the queue raises this fraction from 80.45% to 99.99% under moderate skew and from 90.86% to 99.99% under severe skew. The larger change is in the number of same-class comparisons. Positives per valid anchor increase from 2.54 to 77.85 under moderate skew and from 4.00 to 138.01 under severe skew, and PAMAP2 shows the same pattern with a near-complete valid-anchor fraction and a similar expansion of positives. On both datasets, severe-skew mini-batches already contain a higher fraction of valid anchors, yet the queue produces the larger accuracy gain under severe skew. The accuracy pattern is therefore better explained by the number of available same-class comparisons than by the valid-anchor fraction alone.

Table 7 examines whether this gain remains when all clients participate in every round. Full participation removes variation in client selection, but it does not increase the comparison set inside a client’s mini-batch.

***StabFL*** has the highest mean accuracy in all four columns. Relative to ***StabFL*** w/o Queue, the queue raises CIFAR-10 accuracy by 1.66 percentage points with partial participation and 2.44 percentage points with full participation. The corresponding gains on PAMAP2 are 4.44 and 4.78 percentage points. The queue gain therefore remains after client-selection variation is removed.

Repeated samples and queue composition. Because the queue persists across local epochs and communication rounds, an earlier representation of the current source sample could otherwise reappear in its comparison set. We therefore track a stable source identifier for each queue entry and exclude entries with the same source identifier as the current anchor from both the positive set and the contrastive denominator.

Before this exclusion, exact same-source positives account for only 0.011–0.013% of positive comparisons on severe-skew CIFAR-10 and 0.039–0.047% on severe-skew PAMAP2. After exclusion, their contribution is zero, while the queue retains an average of 115.83 and 122.47 unique-source positives per valid anchor on CIFAR-10 and PAMAP2, respectively. In a matched diagnostic comparison, the duplicate-safe queue remains 2.15 and 9.30 percentage points above the batch-only variant in macro-F1 on the two datasets.

We also examine whether the FIFO queue becomes dominated by locally frequent classes. Under severe CIFAR-10 label skew, the queue class occupancy generally follows each client’s local class distribution. Even for client–class pairs representing less than 5% of the local samples, more than 99.8% of anchors retain at least one valid same-class positive after excluding exact same-source entries. Class-wise recall changes are not uniformly positive across classes, so we do not interpret the queue as guaranteeing minority- class or uniform class-wise improvements. Instead, these results show that its benefit is not explained by exact self-repeated positives or by a complete absence of same-class comparisons for locally rare classes.

#### 6.3.2. Component Effects and Interactions

To examine whether the performance of ***StabFL*** results from independent component gains or from their configuration-dependent interactions, Table 8 compares eight configurations formed by local logit adjustment (LA), full-model proximal regularization (Prox), and queue-augmented supervised contrastive learning (Queue-SupCon). We use CIFAR-10 under severe label skew with α=0.1, where heterogeneous local updates are most pronounced. Disabling Queue-SupCon removes both the supervised contrastive term and its queue. Section 6.3.1 compares batch-only and queue-augmented SupCon to isolate the queue contribution.

Prox produces the largest descriptive average effect. Averaged over the other component settings, enabling Prox increases mean macro-F1 from 56.11% to 70.97%, corresponding to a gain of 14.86 percentage points. The average gains of LA and Queue-SupCon are 3.49 and 3.48 percentage points, respectively. Thus, proximal stabilization produces the largest change among the three components under severe skew.

The effect of LA depends strongly on whether Prox is present. Without Prox or Queue-SupCon, adding LA changes macro-F1 from 54.57% to 52.50%, a decrease of 2.07 percentage points. When Prox is enabled without Queue-SupCon, adding LA instead increases macro-F1 from 66.28% to 73.86%, a gain of 7.58 percentage points. A similar pattern appears when Queue-SupCon is enabled, where the gain from LA increases from 0.49 percentage points without Prox to 7.94 percentage points with Prox. This result is consistent with the analysis in Section 4.4, in which client-specific classification gradients affect the shared encoder and benefit from constraining the complete trainable model.

Queue-SupCon increases the mean macro-F1 in all four matched configurations, although its gain varies from 1.62 to 6.43 percentage points depending on the other components. In the core LA + Prox configuration, adding Queue-SupCon improves macro-F1 from 73.86% to 75.84%, a gain of 1.98 percentage points, and the full configuration achieves the highest mean performance in the table. These results do not indicate uniform and independent gains from every component. Instead, they support a configuration-dependent interpretation in which Prox provides the dominant stabilization effect, LA benefits substantially from that stabilization, and Queue-SupCon further enriches the local representation-learning signal.

### 6.4. Sensitivity Analysis

Finally, we examine sensitivity to label skew, local batch size, queue capacity, reset robustness, and reset frequency. Figure 5a varies label skew, Figure 5b varies the local batch size, Table 9 compares four queue capacities.

#### 6.4.1. Label Skew and Local Batch Size

If stabilized logit adjustment mainly helps when clients have different class distributions, its margin should be small near the balanced setting and increase as label skew becomes stronger. Figure 5a follows this pattern. At α=1.0, LA + Prox and ***StabFL*** reach 88.63% and 88.61%, and the margin over FedProx is 0.78 percentage points. The margin increases to 3.08 percentage points at α=0.3 and 4.44 percentage points at α=0.1.

The queue should also matter less when a larger mini-batch already provides more same-class samples. Figure 5b shows that the queue raises mean accuracy by 0.39 percentage points over ***StabFL*** w/o Queue at batch size 8. At batch size 32, the difference is 0.30 percentage points, and LA + Prox, ***StabFL*** w/o Queue, and ***StabFL*** lie within 0.43 percentage points. This smaller gap agrees with the comparison-set analysis in Figure 4.

#### 6.4.2. Queue Capacity

Table 9 reports the queue-capacity sensitivity under severe CIFAR-10 label skew. Increasing *M* from 32 to 256 raises mean macro-F1 from 74.58% to 75.84%, while M=512 reaches 75.36%. Thus, M=256 achieves the highest mean among the evaluated queue-capacities.

The maximum difference across the four capacities is 1.26 percentage points, while the results for M=128–512 remain within 0.74 percentage points. Therefore, we can identify that performance remains relatively stable across the evaluated range and does not improve monotonically with capacity. We use M=256 as a practical default because it achieves the highest mean, without treating it as a uniquely optimal capacity. Although a larger queue can retain older representations, the small reduction at M=512 does not by itself establish representation staleness as its cause.

#### 6.4.3. Reset Robustness and Frequency

We compare the current encoder-parameter cosine reset with no reset, clearing the queue at every client participation, a projection-feature reset, and a parameter rule applied to both the encoder and projection head. The comparison is conducted under severe CIFAR-10 label skew.

For the projection-feature control, we compute the mean cosine similarity between the normalized projection outputs of the previous local model and the incoming global model on a fixed client-local probe set. Its threshold is selected on the validation tuning seed and fixed at $\delta_{f}=0$ for evaluation. Because these projected representations depend on both the encoder and projection head, this control also captures projection-head drift.

As shown in Table 10, the current encoder-parameter rule achieves the highest mean macro-F1 among the evaluated alternatives. No reset and resetting at every participation reach 74.58% and 74.88%, respectively. The projection-feature control reaches 75.50%, while extending the parameter vector to include the projection head reaches 75.12%. Neither alternative produces a higher mean than the current encoder-only rule at 75.84%. These results support the current rule as a practical choice, but they do not establish parameter cosine similarity itself as a direct measure of feature compatibility.

We also vary the parameter-cosine threshold. Thresholds of δ=0.90, 0.95, and 0.99 produce mean macro-F1 scores of 74.30%, 75.84%, and 74.68%, respectively. Therefore, we retain δ=0.95 as the fixed setting used in the other experiments rather than treating it as a universally optimal threshold.

We define the reset frequency as the fraction of reset checks that result in clearing the queue. With the encoder-parameter rule and δ=0.95, the average reset rates are 41.67% under moderate CIFAR-10 skew, 39.20% under severe CIFAR-10 skew, 5.98% under severe PAMAP2 skew, and 0.54% in the natural-client PAMAP2 evaluation. Therefore, the rule produces substantially different reset behavior across the evaluated settings rather than approximating either no reset or resetting at every participation.

### 6.5. Computation, Memory, and Communication Overhead

Table 11 compares the resource requirements of FedAvg, FedProx, batch-only SupCon, ***StabFL*** w/o Queue, and ***StabFL***. Runtime and peak allocated GPU memory are measured using the CIFAR-10 microbenchmark with batch size B=8 and normalized to FedAvg. Batch SupCon applies cross-entropy and supervised contrastive learning using only the current mini-batch. ***StabFL*** w/o Queue additionally includes local logit adjustment and full-model proximal regularization but uses the same batch-only comparison set. This comparison separates the cost of the complete local objective from the incremental cost of the queue.

The complete ***StabFL*** objective requires 2.44× the runtime of FedAvg, compared with 2.34× for ***StabFL*** w/o Queue. FedProx and batch-only SupCon require 1.70× and 1.68×, respectively. The queue-specific cost is substantially smaller than the cost of the complete local objective. Relative to ***StabFL*** w/o Queue, adding the queue increases the average mini-batch time from 5.570 to 5.804 ms, corresponding to 1.042×, or approximately 4.2%. Peak allocated GPU memory increases by 0.13 MiB, from 90.54 to 90.67 MiB, corresponding to approximately 0.14%. Therefore, both variants appear as 1.04× FedAvg after rounding in Table 11.

With B=8 and M=256, the dense similarity matrix increases from 8×8 for batch-only SupCon to 8×(8+256) for queue-augmented SupCon. The corresponding number of similarity dot products increases from 64 to 2112 per mini-batch. With projected dimension d=128 and approximately 2d floating-point operations per dot product, the similarity calculation increases from approximately 0.016 to 0.541 MFLOPs. This 33× factor applies only to the similarity calculation and should not be interpreted as a 33× increase in end-to-end training cost. The measured queue-specific runtime increase is instead approximately 4.2%, because the complete local update also includes encoder and projection-head computation, backpropagation, and optimizer operations.

The additional persistent local state of ***StabFL*** consists of the queue and the previous encoder retained for the reset check. Their combined sizes are 2.42 MiB for CIFAR-10 and 0.50 MiB for PAMAP2. In the revised duplicate-safe implementation, the queue itself occupies exactly 132 KiB per client (130 KiB for the representations and labels, plus 2 KiB for the stable source identifiers). These values are separate from the transient GPU memory used during local training.

The queue tensors themselves add no network traffic because their representations and labels remain on the client. The method-required bidirectional model payload per selected client and communication round is 4,869,944 bytes for FedAvg and 5,136,416 bytes for ***StabFL*** on CIFAR-10. The corresponding values on PAMAP2 are 805,410 and 1,071,626 bytes. Thus, the normalized communication costs of ***StabFL*** are 1.05× and 1.33×, respectively. This increase results from transmitting the projection head as part of the model state rather than from transmitting the queue.

## 7. Discussion

Local logit adjustment and proximal regularization should be designed jointly. Logit adjustment (LA) incorporates the local class prior at the output logits, but its class-dependent gradients propagate through both the classifier and the feature encoder. Its effect is therefore not confined to the decision layer. This interaction becomes particularly important under severe label skew, where combining LA with full-model proximal regularization improves accuracy by 19.02 percentage points and outperforms the classifier-only and split regularization alternatives. The parameter analysis provides a consistent explanation. Constraining only the classifier minimizes its movement but allows the encoder to undergo a larger client-specific change. In contrast, full-model regularization limits changes along all gradient paths involved in the joint objective, thereby stabilizing both the representation space and the corresponding decision boundaries. This result indicates that the scope of stabilization should follow the components affected by a client-specific objective rather than being selected independently of that objective.

The client-local queue provides a richer and more reliable contrastive signal. Batch-only supervised contrastive learning relies on the class composition of each individual mini-batch. Under small and label-skewed batches, this composition can be highly variable, particularly for classes that occur infrequently on a client. Across the four partial-participation conditions, batch-only SupCon provides no consistent improvement over LA + Prox and performs worse under severe PAMAP2 skew. The client-local queue instead reuses labeled projected representations observed during earlier local updates, allowing the current batch to benefit from a broader history of local observations. The comparison-set statistics show that its primary effect is not simply to make more anchors valid. Rather, it substantially expands the same-class comparison set available to already valid anchors. This distinction is important because a single positive may technically activate the contrastive loss, whereas a richer comparison set provides a more reliable representation-learning signal.

The queue introduces modest practical overhead for mobile and wearable devices. Sensor observations are commonly collected as temporally separated windows, and classes associated with a user’s activities may appear in short or irregular bursts. A small mini-batch may therefore provide only a narrow view of the classes previously observed by the device. The queue converts this locally accumulated history into a reusable comparison set without transmitting raw samples, projected representations, labels, or source identifiers. The queue itself occupies 132 KiB of local storage per client and adds no network traffic. The complete persistent local state additionally includes the previous encoder retained for the reset check, while the communication overhead of ***StabFL*** results from transmitting the projection head rather than the queue. As quantified in Section 6.5, adding the queue to ***StabFL*** w/o Queue increases the average mini-batch time by approximately 4.2% and peak allocated GPU memory by 0.13 MiB in the CIFAR-10 microbenchmark. Queue capacity directly controls the number of additional similarity comparisons and therefore provides a mechanism for adjusting the computation–memory trade-off. These configurable resource requirements are relevant to green computing because queue capacity can be adapted to different device budgets without transmitting the queue contents. However, the measurements characterize resource overhead in our evaluation environment and do not establish energy savings. Deployment performance and energy consumption on actual mobile and wearable hardware remain to be evaluated.

The value of local memory depends on the comparisons already available in each mini-batch. The queue gain remains under full participation, where variability caused by client selection is removed, indicating that its benefit does not arise only from participation randomness. In contrast, the gain becomes smaller when the local batch size increases to 32. Larger batches already contain more same-class comparisons and therefore reduce the marginal value of stored representations. The queue is thus most useful in the operating regime that motivates the method, where resource constraints require small local batches and label skew limits the availability of positive pairs. This observation also suggests that queue capacity need not be fixed uniformly. Devices with larger feasible batch sizes may use a smaller queue, whereas devices operating with very small batches may benefit from a larger or class-balanced memory.

Persistent local memory introduces state-management and representation-staleness challenges. The current design assumes that each client can maintain its identity and local state across communication rounds. A returning device must recover its own queue, which may be difficult after device replacement, application reinstallation, or local storage cleanup. Long participation gaps may also make stored representations less compatible with the current encoder, especially when the global model or the client’s class distribution changes rapidly. Increasing queue capacity provides more comparisons but can also retain older and potentially stale representations for longer. The reset rule mitigates this issue by discarding the queue when the mismatch becomes sufficiently large, although the reset threshold and queue capacity jointly determine how much useful history is preserved and how quickly the comparison set can be rebuilt. Future work can investigate participation-aware expiration, class-balanced queue allocation, adaptive capacity selection, and lightweight compression [27]. Privacy-aware retention policies may also determine how long local entries should remain available even when they are never transmitted outside the device.

The client-local queue does not provide a formal privacy guarantee. The queue follows a local-state-only design in which projected representations, labels, and source identifiers remain on the originating client and are not directly transmitted to the server or other clients. The local class priors are likewise not directly transmitted. This design reduces the information explicitly included in the communication payload, but it should not be interpreted as a formal privacy guarantee. Persistent labeled representations and source identifiers may retain information about local samples and could be exposed if the client device or its storage is compromised. Moreover, information about local data or class distributions may still be inferred from the transmitted model updates [28]. ***StabFL*** does not employ a formal threat model, differential privacy, or secure aggregation because the present study focuses on local training under label skew rather than privacy protection. Accordingly, our privacy-related claim is limited to the non-transmission of queue contents and local class priors. Protecting persistent local state and model updates would require complementary mechanisms such as device-level access control, storage encryption, secure aggregation, or differential privacy.

## 8. Conclusions

We presented ***StabFL***, which combines local logit adjustment, full-model proximal regularization, and queue-augmented supervised contrastive learning for federated learning under label skew. Full-model regularization stabilizes the encoder and classifier jointly with its benefit becoming more pronounced under severe skew. The client-local queue enriches same-class comparisons beyond the current mini-batch and yields higher mean performance than the batch-only variant in the evaluated CIFAR-10 and PAMAP2 settings. ***StabFL*** achieves the highest mean accuracy among the evaluated baselines in all four partial-participation conditions. The queue gain remains under full participation but decreases with a larger batch size, showing that it is most useful when local comparisons are limited.

## Figures and Tables

**Figure 1:**
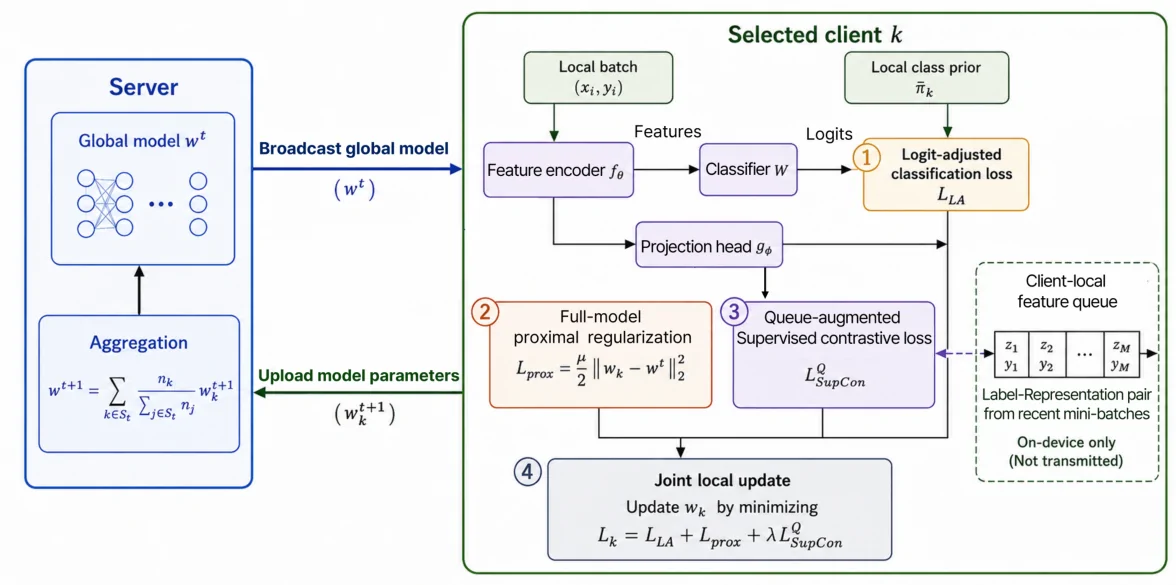
Overview of ***StabFL***. Each selected client evaluates logit-adjusted classification, full-model proximal regularization, and supervised contrastive regularization with a client-local queue as one joint local objective. The server broadcasts the current global model and aggregates only updated model parameters. Local class priors, queued representations, and labels remain on the client.

**Figure 2:**
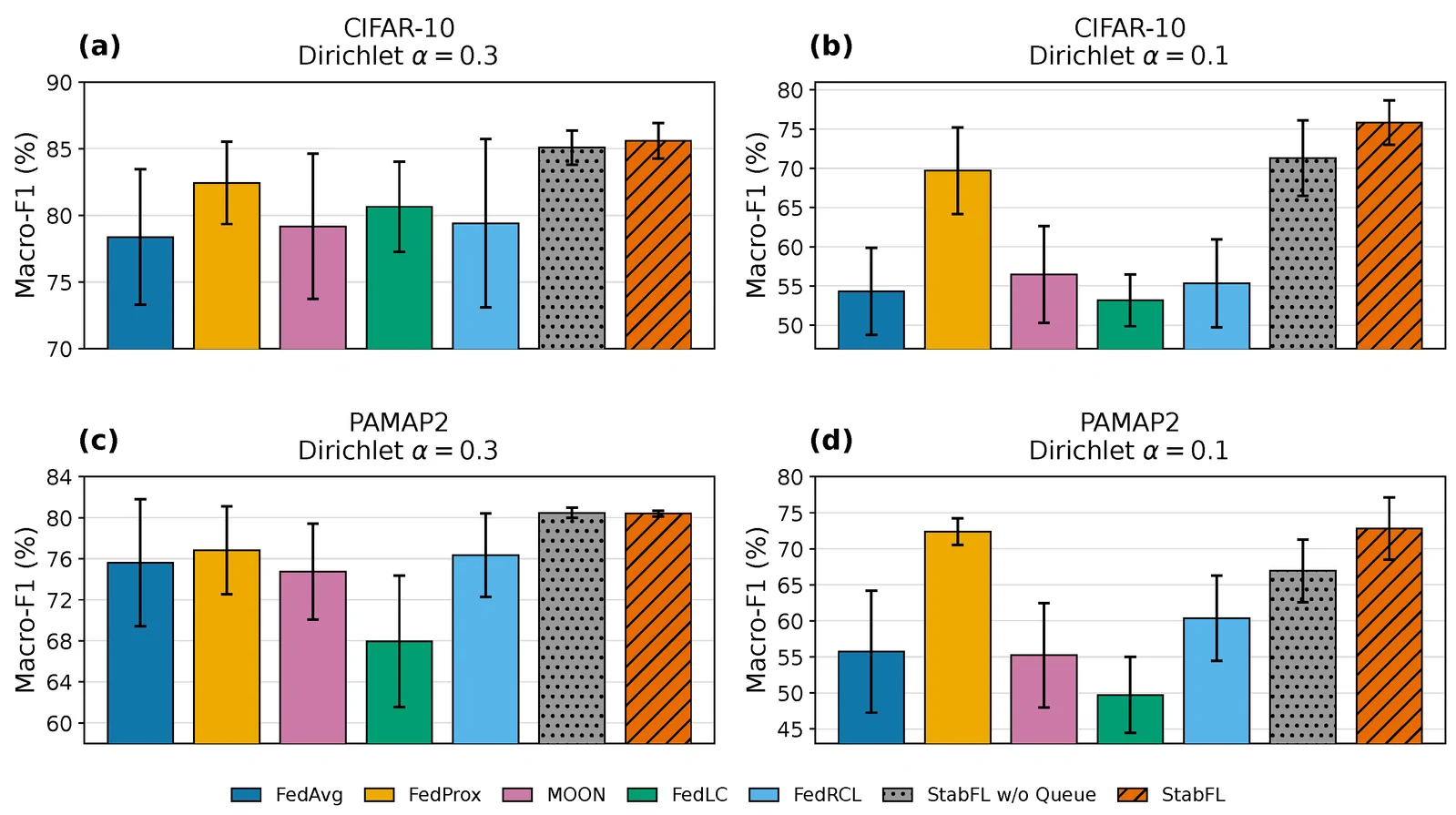
Global test macro-F1 of the main baselines under partial participation with five of 20 clients selected per round. Panels (**a**,**b**) report CIFAR-10 at α=0.3 and α=0.1. Panels (**c**,**d**) report PAMAP2 under the same skew levels.

**Figure 3:**
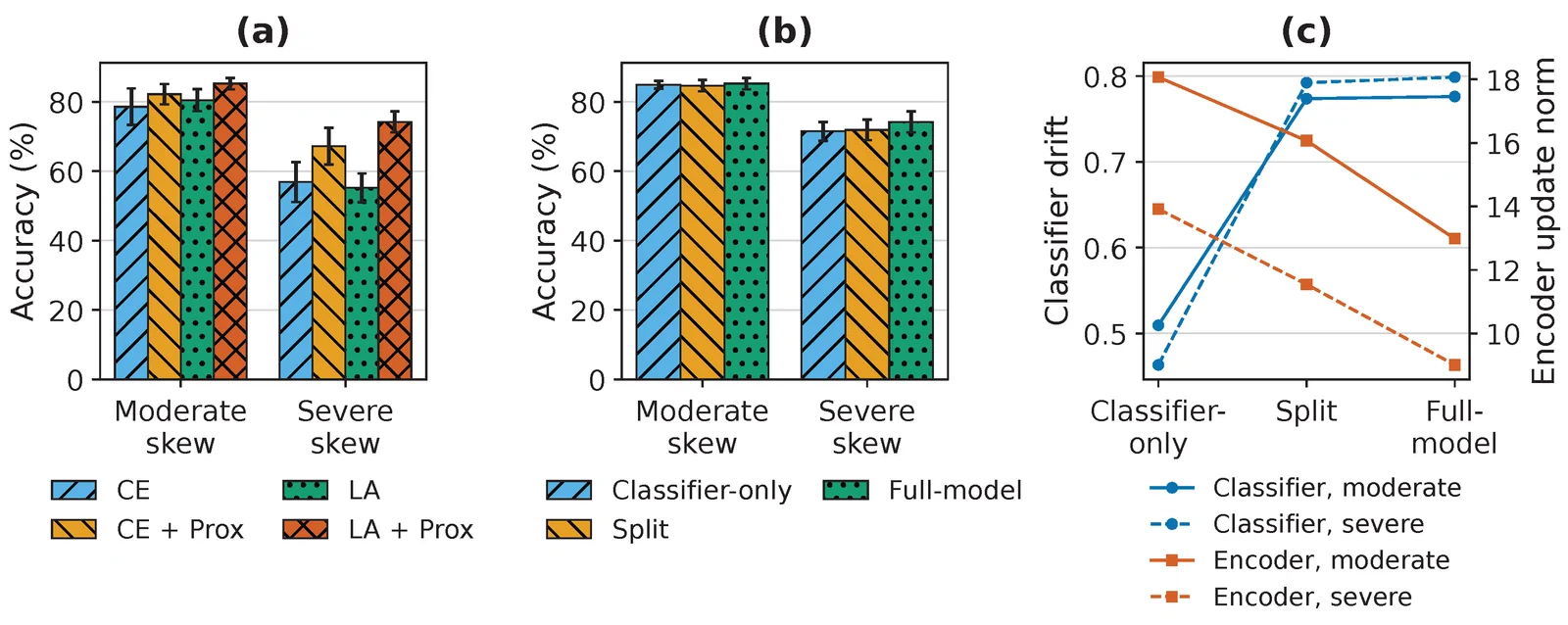
Analysis of LA with proximal regularization on CIFAR-10 under partial participation with batch size 8. (**a**) Accuracy for cross-entropy and LA with and without full-model proximal regularization. (**b**) Accuracy under classifier-only, split, and full-model proximal scopes. (**c**) Classifier drift and encoder update norms for the same scopes under moderate and severe skew. Error bars in the accuracy panels show standard deviation.

**Figure 4:**
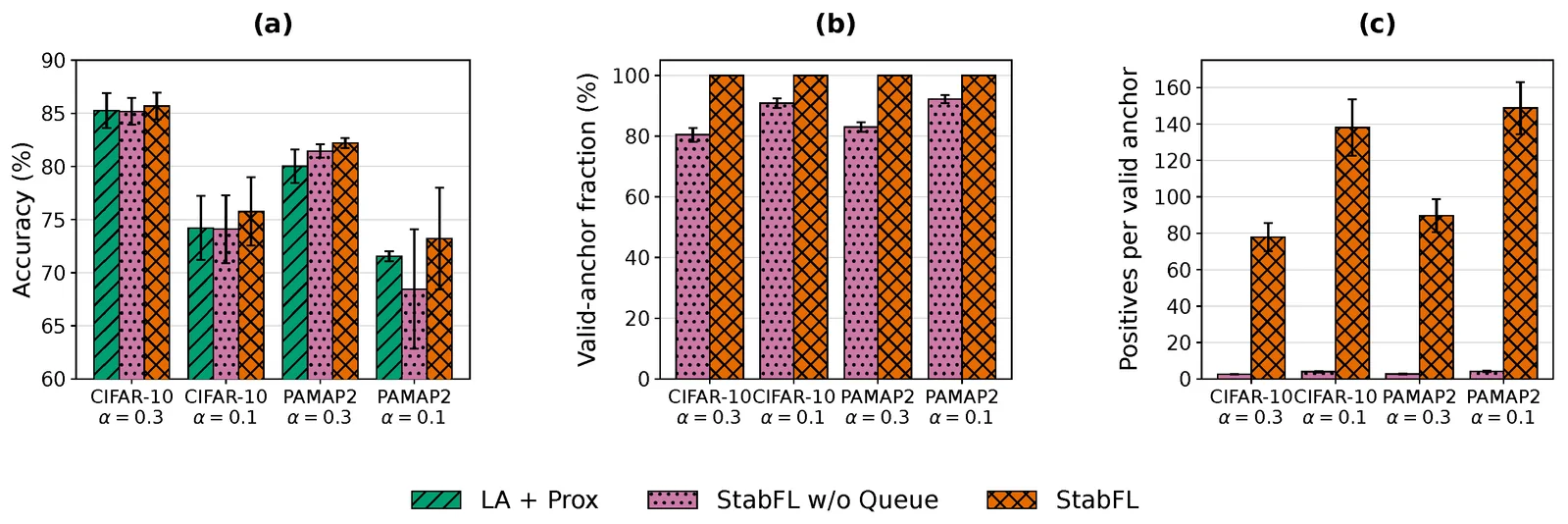
Contribution of the client-local queue under partial participation. (**a**) Accuracy for LA + Prox, ***StabFL*** w/o Queue, and ***StabFL*** on CIFAR-10 and PAMAP2. (**b**) Fraction of anchors with at least one positive. (**c**) Positives per valid anchor. Bars show the mean, and error bars show standard deviation.

**Figure 5:**
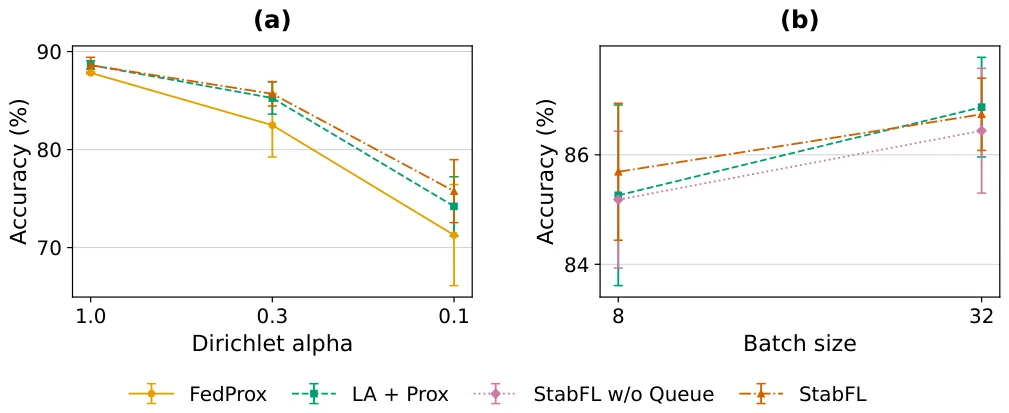
Accuracy sensitivity on CIFAR-10. (**a**) Label skew with 5/20 clients and batch size 8. (**b**) Batch size at α=0.3 with 5/20 clients. Error bars show standard deviation.

**Table 1:** Methodological and implementation characteristics of StabFL and related methods.

Method	Main Objective	Treatment of Label Skew	
FedLC	Calibrated classification	Class-frequency-based logit calibration	
FedRS	Restricted-softmax classification	Restricts updates associated with locally missing classes	
FedProx	Classification + proximal penalty	Constrains heterogeneous local updates	
MOON	Classification + model contrast	Aligns local representations with the global model	
FedProto	Classification + prototype alignment	Aligns class representations across clients	
FedPCL	Prototype-wise contrastive learning	Transfers class-level information across clients	
FedRCL	Classification + relaxed contrastive loss	Limits within-class representation collapse	
DCFL (sample-wise)	Classification + decoupled alignment and uniformity	Separately controls attraction and repulsion under heterogeneity	
Cross-Batch Memory	Memory-augmented metric learning	No FL-specific label-skew treatment	
StabFL (Ours)	Logit-adjusted classification + full-model Prox + queue-SupCon	Local-prior correction with stabilized updates and expanded comparisons	
Method	Communicated information	Client-local memory	Computational requirement
FedLC	Model parameters	Local class statistics	Class-dependent logit calibration
FedRS	Model parameters	Local class-support information	Class-dependent logit scaling
FedProx	Model parameters	Global parameter reference	Parameter-distance penalty
MOON	Model parameters	Global and previous local models	Reference-model forward passes and similarity calculations
FedProto	Class prototypes	Local and global prototypes	Prototype computation and alignment
FedPCL	Class prototypes	Fixed pretrained models and prototype sets	Pretrained feature extraction and prototype comparisons
FedRCL	Model parameters	No cross-batch representation memory	Within-batch pairwise similarities and divergence penalty
DCFL (sample-wise)	Model parameters	No cross-batch representation memory	Within-batch pairwise similarities and separate loss terms
Cross-Batch Memory	Not applicable (centralized)	Historical embeddings and labels	Batch-to-memory similarities and memory updates
StabFL (Ours)	Model parameters only	Labeled FIFO queue, global reference, and previous encoder state	Logit adjustment, proximal penalty, batch-to-queue similarities, and reset check

**Table 2:** Key design elements of ***StabFL*** and related methods. “—” denotes an element not explicitly included in the original formulation.

Method	Prior Correction	Proximal Constraint	Contrastive Learning	Cross-Batch Memory
FedLC	Calibration	—	—	—
FedRS	Restricted softmax	—	—	—
FedProx	—	Full-model	—	—
MOON	—	—	Model-level	—
FedProto	—	—	—	—
FedPCL	—	—	Prototype-wise	—
FedRCL	—	—	Relaxed sample-level	—
DCFL (sample-wise)	—	—	Decoupled sample-level	—
Cross-Batch Memory	—	—	Pair-based	Embedding memory
StabFL (Ours)	Log-prior	Full-model	Supervised sample-level	Client-local queue

**Table 3:** Dataset and training configuration.

Dataset	Classes	Clients	Input	Partition	Rounds	
CIFAR-10	10	20	3×32×32	Dirichlet α=0.3	100	
CIFAR-10	10	20	3×32×32	Dirichlet α=0.1	200	
PAMAP2	12	20	18×128	Dirichlet α=0.3	100	
PAMAP2	12	20	18×128	Dirichlet α=0.1	200	
Dataset	Local epochs	Batch	Optimizer	Learning rate	Weight decay	Augmentation
CIFAR-10	5	8	Adam	$10^{-3}$	$10^{-4}$	Flip, reflection pad, random crop
PAMAP2	5	8	Adam	$10^{-3}$	$10^{-4}$	None

**Table 4:** Performance comparison under moderate (α=0.3) and severe (α=0.1) label skew.

Method	CIFAR-10				PAMAP2			
	α=0.3		α=0.1		α=0.3		α=0.1	
	Accuracy	Macro-F1	Accuracy	Macro-F1	Accuracy	Macro-F1	Accuracy	Macro-F1
FedAvg	78.58±5.27	78.38±5.08	57.89±4.42	54.31±5.54	76.27±5.58	75.61±6.18	59.77±8.92	55.73±8.47
FedProx	82.49±3.25	82.44±3.09	71.32±3.67	69.70±5.52	76.90±4.21	76.81±4.29	73.87±3.53	72.37±1.84
MOON	79.20±5.63	79.18±5.44	59.04±4.51	56.48±6.17	74.91±3.89	74.75±4.69	60.10±8.89	55.23±7.23
FedLC	80.75±3.12	80.65±3.37	57.34±3.13	53.16±3.30	71.60±6.45	67.94±6.42	56.95±7.39	49.73±5.29
FedRCL	79.65±6.36	79.41±6.30	59.70±4.97	55.34±5.61	77.34±3.54	76.35±4.06	64.99±6.53	60.38±5.93
***StabFL*** w/o Queue	85.18±1.25	85.08±1.28	74.10±3.20	71.31±4.80	81.45±0.64	80.47±0.50	70.24±4.83	66.94±4.34
***StabFL***	85.57±1.36	85.59±1.33	75.76±3.21	75.84±2.83	83.21±0.46	82.42±0.28	74.68±4.10	72.82±4.32

The best result is shown in bold.

**Table 5:** PAMAP2 evaluation with natural subject boundaries. Values are mean ± standard deviation across the nine held-out subjects.

Method	Macro-F1 (%)
FedAvg	82.59±2.10
FedProx	82.45±0.93
FedLC	81.86±0.74
FedRCL	80.60±3.44
***StabFL*** w/o Queue	81.93±0.76
***StabFL***	85.33±0.80

The best result is shown in bold.

**Table 6:** Matched-coefficient comparison for proximal regularization under severe CIFAR-10 label skew (α=0.1).

μ	Classifier Only	Encoder + Projector	Full Model
$3\times 10^{-4}$	64.45±2.54	58.49±3.71	66.87±1.54
$1\times 10^{-3}$	68.61±4.26	62.06±4.07	73.86±2.87
$3\times 10^{-3}$	71.02±2.85	63.91±5.86	78.15±1.87

The best result is shown in bold.

**Table 7:** Global test accuracy (%) under severe label skew with α=0.1 for partial and full participation.

Method	CIFAR-10		PAMAP2	
	5/20	20/20	5/20	20/20
FedProx	71.32±3.67	71.86±7.31	73.87±3.53	72.09±3.93
FedLC + Prox	73.30±2.53	74.30±3.42	69.76±1.80	66.38±4.54
LA + Prox	74.21±3.02	74.06±2.13	71.55±0.48	67.11±7.27
***StabFL*** w/o Queue	74.10±3.20	72.91±2.64	70.24±4.83	69.26±7.16
***StabFL***	75.76±3.21	75.35±3.60	74.68±4.10	74.04±6.01

The best result is shown in bold.

**Table 8:** Component analysis under severe CIFAR-10 label skew (α=0.1).

LA	Prox	Queue-SupCon	Macro-F1	Δ vs. Full (pp)
No	No	No	54.57±7.68	−21.27
No	No	Yes	58.44±11.45	−17.40
No	Yes	No	66.28±7.03	−9.56
No	Yes	Yes	67.90±7.88	−7.94
Yes	No	No	52.50±4.26	−23.34
Yes	No	Yes	58.93±6.96	−16.91
Yes	Yes	No	73.86±2.87	−1.98
Yes	Yes	Yes	75.84±2.83	0.00

The best result is shown in bold.

**Table 9:** Queue-capacity sensitivity under severe CIFAR-10 label skew.

Queue Capacity *M*	Macro-F1
32	74.58±4.99
128	75.10±3.10
256	75.84±2.83
512	75.36±2.88

The best result is shown in bold.

**Table 10:** Comparison of queue-reset strategies under severe CIFAR-10 label skew.

Reset Strategy	Macro-F1 (%)
No reset	74.58±3.42
Reset at every participation	74.88±2.99
Projection-feature	75.50±2.40
Encoder + projector parameter reset	75.12±2.35
Encoder-parameter reset (δ=0.95)	75.84±2.83

The best result is shown in bold.

**Table 11:** Relative computation, memory, communication, and persistent local-state overhead.

Method	Runtime	Peak GPU mem.	Comm. (CIFAR)	Comm. (PAMAP2)	Extra Persistent Local State
FedAvg	1.00×	1.00×	1.00×	1.00×	–
FedProx	1.70×	1.04×	1.00×	1.00×	–
Batch SupCon	1.68×	1.00×	1.05×	1.33×	–
***StabFL*** w/o Queue	2.34×	1.04×	1.05×	1.33×	–
***StabFL***	2.44×	1.04×	1.05×	1.33×	2.42/0.50 MiB ^†^

^†^ Queue plus retained previous encoder for CIFAR-10/PAMAP2. The queue itself uses exactly 132 KiB per client, including 128 KiB for representations, 2 KiB for labels, and 2 KiB for stable source identifiers. Queue contents are never transmitted. Methods using the projection head transmit the projector as part of the model state.

## Data Availability

Publicly available datasets were analyzed in this study. CIFAR-10 is available at https://www.cs.toronto.edu/~kriz/cifar.html (accessed on 19 September 2026), and PAMAP2 is available at https://archive.ics.uci.edu/dataset/231/pamap2+physical+activity+monitoring (accessed on 19 September 2026). The source code, configuration files, and hyperparameter settings used in this study are available at https://github.com/MakerDev/stabfl (accessed on 19 September 2026).
